# Crystal structure of a four-layered [3.3](3,5)pyridino­phane

**DOI:** 10.1107/S1600536814023691

**Published:** 2014-10-31

**Authors:** Masahiko Shibahara, Motonori Watanabe, Taisuke Matsumoto, Kenta Goto, Teruo Shinmyozu

**Affiliations:** a Department of Chemistry, Faculty of Education and Welfare Science, Oita University, 700 Dannoharu, Oita 870-1192, Japan; b International Institute for Carbon-Neutral Energy Research (I2CNER), Kyushu University, 744 Motooka, Nishi-ku, Fukuoka 819-0395, Japan; c Evaluation Center of Materials Properties and Function, Institute for Materials Chemistry and Engineering (IMCE), Kyushu University, 6-1 Kasuga-koen, Kasuga-city, Fukuoka 816-8580, Japan; d Institute for Materials Chemistry and Engineering (IMCE), Kyushu University, 6-10-1 Hakozaki, Higashi-ku, Fukuoka 812-8581, Japan

**Keywords:** crystal structure, meta­cyclo­phane, pyridino­phane, transannular *p*-electronic inter­action, C—H⋯N hydrogen bond, inter­molecular short contact

## Abstract

The title compound, C_40_H_46_N_2_ {systematic name: 12,30-di­aza­hepta­cyclo[21.13.1.1^5,19^.1^6,18^.1^10,14^.1^24,36^.1^28,32^]do­tetra­conta-1(37),5(40),6(41),10(42),11,13,18,23,28,30,32(39),36(38)-dodeca­ene}, has *syn*–*anti*–*syn* geometry wherein the two outer [3.3]meta­cyclo­phane (MCP) moieties have a *syn* geometry, and contain the facing benzene and pyridine rings at dihedral angles of 26.26 (10) and 26.46 (10)°, respectively. The rings of the central [3.3]MCP unit are not parallel, but orientated at a slight angle of 2.66 (9)°. Three bridging methyl­ene groups are disordered over two sets of sites in a 0.60:0.40 ratio. In the crystal, the mol­ecules are linked by C—H⋯N inter­actions and inter­molecular C—H⋯π short contacts, generating a three-dimensional network.

## Abstract 2

The title compound {systematic name: 12,30-di­aza­hepta­cyclo­[21.13.1.1^5,19^.1^6,18^.1^10,14^.1^24,36^.1^28,32^]do­tetra­conta-1(37),5(40),6(41),10 (42),11,13,18,23,28,30,32 (39),36 (38)-dodeca­ene} has *syn*–*anti*–*syn* geometry. Two types of inter­molecular short contacts are observed in its crystal structure.

## Chemical context   

[3.3]Meta­pyridino­phanes (MPyPs) have been used as ligands in transition metal complexes, and various kinds of metal complexes have been prepared using them (Muralidharan *et al.*, 1989 ▶; Fronczek *et al.*, 1989 ▶; Krüger, 1995 ▶). A variety of types of [3.3]MPyPs are possible, and the [3.3](2,6)PyPs have been studied in detail (Vögtle & Schunder, 1969 ▶; Shinmyozu *et al.*, 1986 ▶; Bottino *et al.*, 1988 ▶). Only a limited number of [3.3](3,5)PyPs have been produced up to now, mainly because of the instability of the coupling precursor, 3,5-bis­(halometh­yl)pyridine. We have previously used freshly prepared 3,5-bis­(chloro­meth­yl)pyridine as the coupling reaction to prepare 2,11-di­aza­[3.3](3,5)PyP (Satou & Shinmyozu, 2002 ▶). One of the major advantages of using [3.3](3,5)PyPs over using [3.3](2,6)PyPs is the potential for forming self-assembled supra­molecules when [3.3](3,5)PyPs become coordinated. This occurs because the meta­cyclo­phanes (MCPs) have *syn* geometries and the nitro­gen lone-pair electrons can readily coordinate with metals without steric hindrance being caused by the bridges. We have also described the synthesis of multilayered [3.3]cyclo­phanes using the (*p*-tolyl­sulfon­yl)methyl isocyanide method (MCPs; Shibahara *et al.*, 2007 ▶) and the (*p*-ethyl­benzene­sulfon­yl)methyl isocyanide method (para­cyclo­phanes; Shibahara *et al.*, 2008 ▶). Multilayered [3.3]MCPs that have a pyridine ring at each end may, therefore, form larger supra­molecules when they form complexes with transition metals. These new types of supra­molecules could have uses as catalysts, inclusion hosts or nanometer-scale materials.
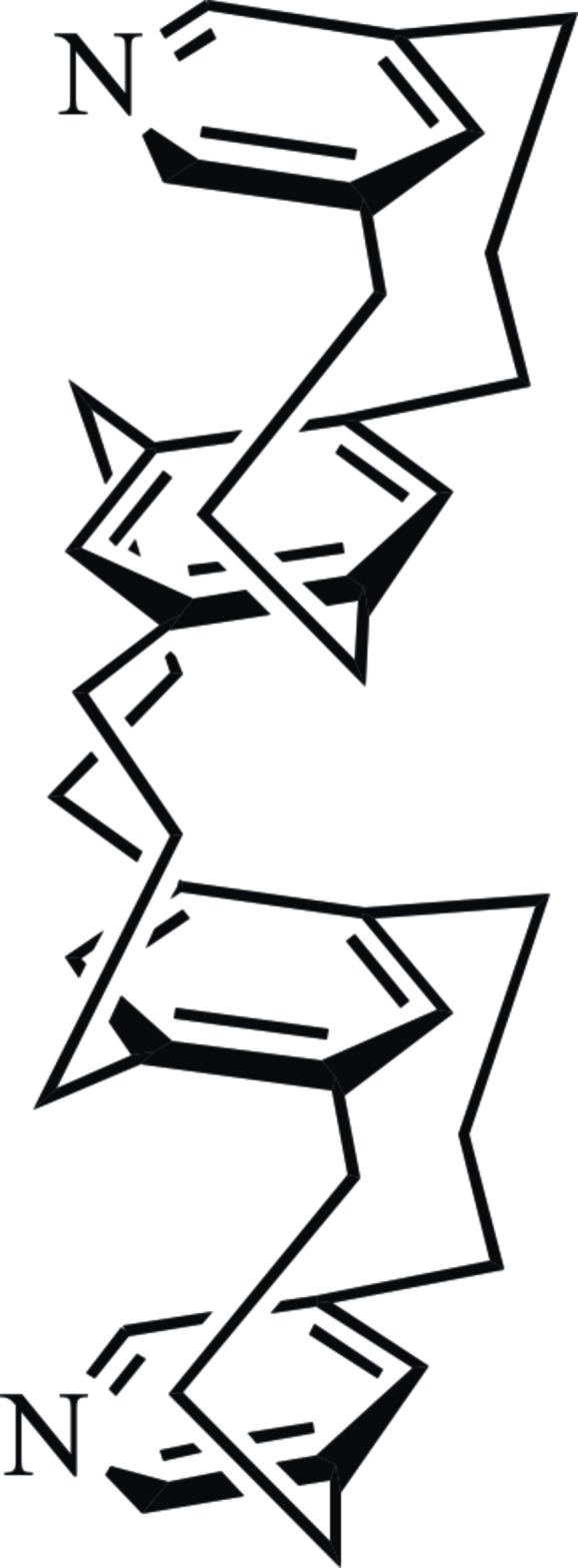


## Structural commentary   

**Figure 1:**
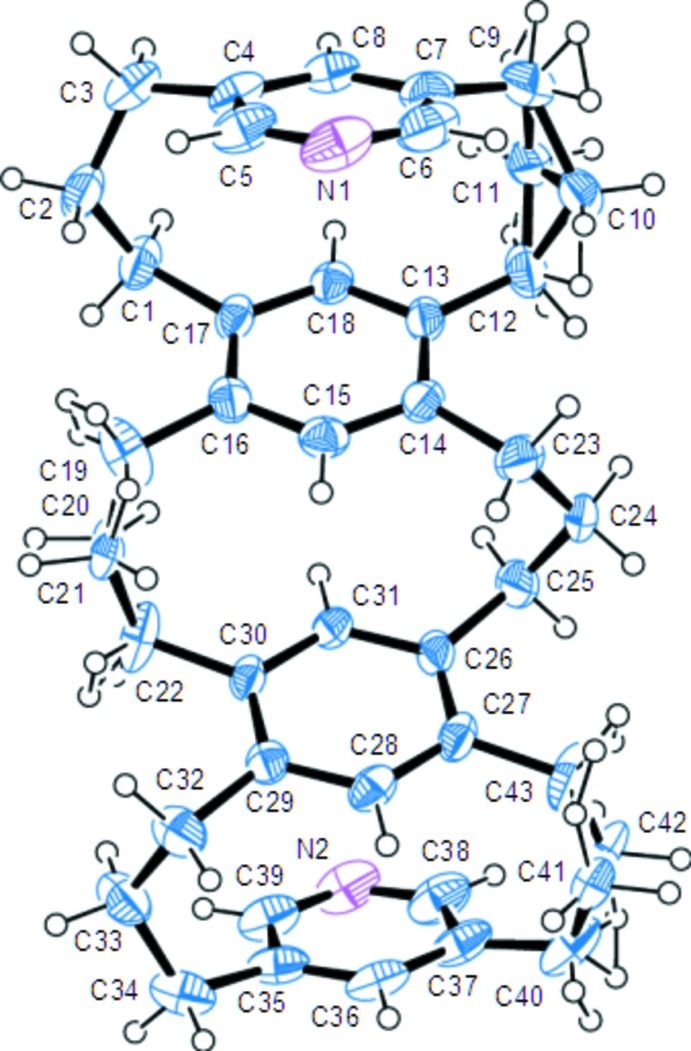
The mol­ecular structure of the title compound, showing the atom-numbering scheme. Displacement ellipsoids are drawn at the 50% probability level.

The mol­ecular structure of the title compound (at 123 K) is shown Fig.1. The tri­methyl­ene bridges are highly flexible and disordered even at this temperature. The mol­ecule has a *syn*–*anti*–*syn* geometry, in which the two outer [3.3]MCP moieties have a *syn* geometry and contain opposing benzene and pyridine rings at angles of 26.26 (10)° (between the C4–C8/N1 and C13—C18 planes) and 26.46 (10)° (between the C26—C31 and C35–C39/N2 planes). These angles are comparable to the corresponding angle (24°) in the parent two-layered [3.3]MCP (Semmelhack *et al.*, 1985 ▶). The central [3.3]MCP unit is not parallel, but is at a slight angle of 2.66 (9)° between the C13–C18 and C26–C31 planes. There is a twist between the benzene rings of the parent two-layered [3.3]MCP of *ca* 15° about the axis through the centre of each ring, but the twists in the outer [3.3]MCP moieties are only 3.93° (between the N1–C8 and C15–C18 axes) and 2.49° (between the C28–C31 and N2–C36 axes), and the benzene rings overlap each other completely in this mol­ecule. However, the twist in the benzene rings in the central [3.3]MCP unit is quite large, at 11.6° between the C15–C18 and C28–C31 axes. The transannular distances between C8 and C18 [2.968 (3) Å], C28 and C36 [2.955 (3) Å], N1 and C15 [4.168 (3) Å], and N2 and C31 [4.174 (3) Å] are comparable to the distances in the parent two-layered [3.3]MCP (2.995 and 4.171 Å) while the distance between C15 and C31 [2.910 (3) Å] is much shorter than that in the parent two-layered [3.3]MCP-2,11-dione (2.99 Å), which adopts an *anti* geometry (Isaji *et al.*, 2001 ▶).

## Supra­molecular features   

**Figure 2:**
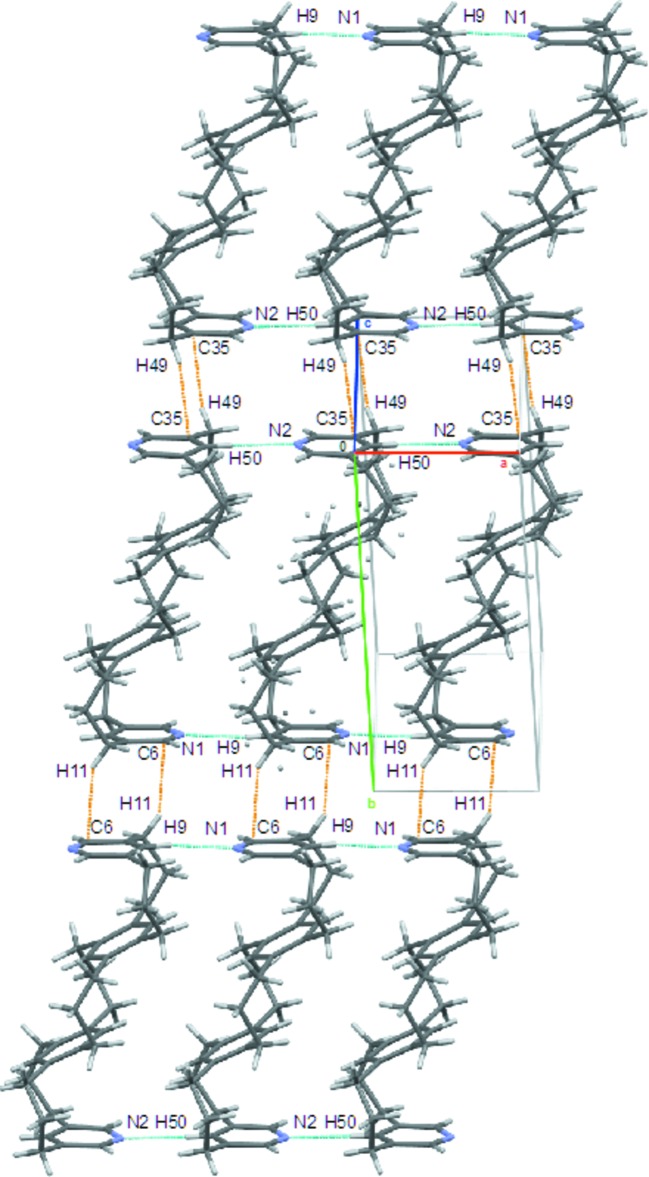
Short contacts of the title compound; C—H⋯π-type inter­actions between C6 and H11 and C35 and H49 (orange dashed lines) and short contacts between N1 and H9 and N2 and H50 (light-blue dashed lines).

The crystal-packing diagram of the mol­ecule (Fig. 2 ▶) shows that mol­ecules are stacked alternately changing direction in the *bc* plane. Two types of inter­molecular short contacts are observed. One is the C—H⋯π-type inter­actions between C6 and H11 (2.811 Å) and between C35 and H49 (2.868 Å) in the *bc* plane, while the other is between N1 and H9 (2.429 Å) and between N2 and H50 (2.468 Å) along the *a* axis (Table 1 ▶). Both instances of the second type of short contact were found to be shorter than the sum of the van der Waals radii of a nitro­gen and hydrogen atom.

## Database survey   

The title compound is closely related to the four-layered [3.3]MCP, hepta­cyclo[21.13.1.1^5,19^.1^6,18^.1^10,14^.1^24,36^.1^28,32^]dotetra­conta-1(37),5(40),6(41),10(42),11,13,18,23,28,30,32(39),36(38)-dodeca­ene), which is the hydro­carbon-only parent mol­ecule (Shibahara *et al.*, 2007 ▶), and its charge-transfer complex with tetra­cyano­ethyl­ene (Shibahara *et al.*, 2011 ▶, 2014 ▶). The four-layered [3.3]MCP changes conformation in the solid state depending on the environment its circumference is in, having a *syn*–*anti*–*syn* geometry like the letter ‘ω’ in a ligand-free environment and have a geometry like the letter ‘s’ when it forms a complex.

## Synthesis and crystallization   

The title compound was prepared as described by Shibahara *et al.* (2008 ▶) by a coupling reaction of 5,7,14,16-tetra­kis­(bromo­meth­yl)[3.3]meta­cyclo­phane with 3,5-bis­[2-iso­cyano-2-(tolyl­sulfon­yl)eth­yl]pyridine, which afforded four-layered [3.3](3,5)pyridino­phane tetra­one, which was converted to the four-layered[3.3](3,5)pyridino­phane Shibahara *et al.*, 2009 ▶) by a Wolff–Kishner reduction. Purification of the crude product by silica gel column chromatography with CH_2_Cl_2_/EtOH (9:1; *v/v*, *R_f_* = 0.53) gave the four-layered pyridino­phane (12% isolated yield in two steps). Finally, the product was crystallized from CH_2_Cl_2_/acetone to give single crystals (colourless prisms), m.p. 518 K (decomposed).

^1^H NMR (600 MHz, CDCl_3_): δ 1.8–2.0 (*m*, 12H, CH_2_C*H*_2_CH_2_), 2.4–2.7 (*m*, 24H, C*H*_2_CH_2_C*H*_2_), 5.97 (*s*, 2H, ArH), 6.21 (*s*, 2H, ArH), 6.91 (*s*, 2H, ArH), 7.84 (*d*, *J* = 1.5 Hz, 4H, ArH). ^13^C NMR (150 MHz, CDCl_3_) δ 26.2, 27.7, 32.4, 32.7, 33.2, 134.0, 134.4, 134.8, 134.8, 135.8, 140.4, 146.8. HRMS (FAB): *m*/*z* [*M*+H]^+^ calculated for C_40_H_47_N_2_ 555.3739, found 555.3739. Analysis calculated for C_40_H_46_N_2_: C, 86.59; H, 8.36; N, 5.05. found: C, 86.35; H, 8.34; N, 5.01.

## Refinement   

Crystal data, data collection and structure refinement details are summarized in Table 2 ▶. H atoms were positioned geom­etrically and refined using a riding model: C—H = 0.95–0.99 Å with *U*_iso_(H) = 1.2*U*_eq_(C).

## Supplementary Material

Crystal structure: contains datablock(s) I. DOI: 10.1107/S1600536814023691/hb7300sup1.cif

Structure factors: contains datablock(s) I. DOI: 10.1107/S1600536814023691/hb7300Isup2.hkl

Supporting information file. DOI: 10.1107/S1600536814023691/hb7300Isup3.cml

CCDC reference: 1031274

Additional supporting information:  crystallographic information; 3D view; checkCIF report

## Figures and Tables

**Table 1:** Hydrogen-bond geometry (, )

*D*H*A*	*D*H	H*A*	*D* *A*	*D*H*A*
C8H9N1^i^	0.95	2.43	3.373(3)	173
C36H50N2^ii^	0.95	2.47	3.394(3)	165

Symmetry codes: (i) 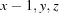; (ii) 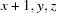.

**Table 2:** Experimental details

Crystal data	
Chemical formula	C_40_H_46_N_2_
*M*_r_	554.79
Crystal system, space group	Triclinic, *P* 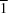
Temperature (K)	123
*a*, *b*, *c* ()	6.1377(15), 14.643(4), 17.519(4)
, , ()	75.619(16), 88.369(17), 86.755(17)
*V* (^3^)	1522.6(7)
*Z*	2
Radiation type	Cu *K*
(mm^1^)	0.52
Crystal size (mm)	0.45 0.30 0.16

Data collection	
Diffractometer	Rigaku R-AXIS RAPID
No. of measured, independent and observed [*I* > 2(*I*)] reflections	20167, 5396, 4455
*R*_int_	0.040
(sin /)_max_ (^1^)	0.602

Refinement	
*R*[*F* ^2^ > 2(*F* ^2^)], *wR*(*F* ^2^), *S*	0.070, 0.204, 1.07
No. of reflections	5396
No. of parameters	410
H-atom treatment	H atoms treated by a mixture of independent and constrained refinement
_max_, _min_ (e ^3^)	0.42, 0.32

Computer programs: *RAPID-AUTO* (Rigaku, 1998 ▶), *SIR2011* (Camalli *et al.*, 2012 ▶), *SHELXL2014* (Sheldrick, 2008 ▶), *Yadokari-XG 2009* (Wakita, 2001 ▶; Kabuto *et al.*, 2009 ▶), *ORTEP-3 for Windows* (Farrugia, 2012 ▶), *Mercury* (Macrae *et al.*, 2006 ▶), *publCIF* (Westrip, 2010 ▶) and *enCIFer* (Allen *et al.*, 2004 ▶).

## supplementary crystallographic information

### Crystal data

**Table:**

C_40_H_46_N_2_	*Z* = 2
*M**_r_* = 554.79	*F*(000) = 600
Triclinic, *P*1	*D*_x_ = 1.210 Mg m^−^^3^
*a* = 6.1377 (15) Å	Cu *K*α radiation, λ = 1.54187 Å
*b* = 14.643 (4) Å	Cell parameters from 20167 reflections
*c* = 17.519 (4) Å	θ = 3.1–68.2°
α = 75.619 (16)°	µ = 0.52 mm^−^^1^
β = 88.369 (17)°	*T* = 123 K
γ = 86.755 (17)°	Block, colorless
*V* = 1522.6 (7) Å^3^	0.45 × 0.30 × 0.16 mm

### Data collection

**Table:**

Rigaku R-AXIS RAPID diffractometer	4455 reflections with *I* > 2σ(*I*)
Radiation source: Rotating anode	*R*_int_ = 0.040
Graphite monochromator	θ_max_ = 68.2°, θ_min_ = 3.1°
Detector resolution: 10.00 pixels mm^-1^	*h* = −7→7
ω scans	*k* = −17→17
20167 measured reflections	*l* = −21→20
5396 independent reflections	

### Refinement

**Table:**

Refinement on *F*^2^	0 restraints
Least-squares matrix: full	Hydrogen site location: inferred from neighbouring sites
*R*[*F*^2^ > 2σ(*F*^2^)] = 0.070	H atoms treated by a mixture of independent and constrained refinement
*wR*(*F*^2^) = 0.204	*w* = 1/[σ^2^(*F*_o_^2^) + (0.0985*P*)^2^ + 0.5512*P*] where *P* = (*F*_o_^2^ + 2*F*_c_^2^)/3
*S* = 1.07	(Δ/σ)_max_ < 0.001
5396 reflections	Δρ_max_ = 0.42 e Å^−^^3^
410 parameters	Δρ_min_ = −0.32 e Å^−^^3^

### Special details

**Table:**

Geometry. All e.s.d.'s (except the e.s.d. in the dihedral angle between two l.s. planes) are estimated using the full covariance matrix. The cell e.s.d.'s are taken into account individually in the estimation of e.s.d.'s in distances, angles and torsion angles; correlations between e.s.d.'s in cell parameters are only used when they are defined by crystal symmetry. An approximate (isotropic) treatment of cell e.s.d.'s is used for estimating e.s.d.'s involving l.s. planes.
Refinement. Refinement was performed using all reflections. The weighted *R*-factor (*wR*) and goodness of fit (*S*) are based on F2. *R*-factor (gt) are based on *F*. The threshold expression of F2 > 2.0 σ (F2) is used only for calculating *R*-factor (gt).

### Fractional atomic coordinates and isotropic or equivalent isotropic displacement parameters (Å^2^)

**Table:**

	*x*	*y*	*z*	*U*_iso_*/*U*_eq_	Occ. (<1)
N1	−0.1497 (3)	0.89284 (14)	0.15093 (15)	0.0710 (6)	
N2	−0.3765 (3)	0.11241 (14)	0.34741 (14)	0.0686 (6)	
C1	−0.6871 (4)	0.71516 (16)	0.33718 (12)	0.0548 (6)	
H1	−0.6992	0.6659	0.3871	0.066*	
H2	−0.8360	0.7317	0.3161	0.066*	
C2	−0.5956 (4)	0.80319 (16)	0.35507 (13)	0.0611 (6)	
H3	−0.6656	0.8121	0.4045	0.073*	
H4	−0.4377	0.7900	0.3652	0.073*	
C3	−0.6234 (4)	0.89604 (15)	0.29260 (14)	0.0568 (6)	
H5	−0.7804	0.9087	0.2805	0.068*	
H6	−0.5757	0.9476	0.3146	0.068*	
C4	−0.4983 (3)	0.89824 (13)	0.21695 (14)	0.0489 (5)	
C5	−0.2702 (3)	0.89796 (15)	0.21396 (16)	0.0593 (6)	
H7	−0.1969	0.9017	0.2600	0.071*	
C6	−0.2568 (4)	0.88533 (16)	0.08720 (17)	0.0694 (7)	
H8	−0.1726	0.8797	0.0421	0.083*	
C7	−0.4813 (4)	0.88527 (14)	0.08282 (14)	0.0585 (6)	
C8	−0.6003 (3)	0.89542 (13)	0.14854 (13)	0.0505 (5)	
H9	−0.7551	0.9006	0.1466	0.061*	
C9	−0.5889 (6)	0.86779 (17)	0.01185 (15)	0.0813 (9)	
H10	−0.4757	0.8638	−0.0288	0.098*	0.6
H11	−0.6909	0.9221	−0.0105	0.098*	0.6
H12	−0.5535	0.9178	−0.0356	0.098*	0.4
H13	−0.7493	0.8692	0.0196	0.098*	0.4
C10	−0.5062 (11)	0.7705 (4)	0.0003 (3)	0.0600 (14)	0.4
H14	−0.5447	0.7669	−0.0533	0.072*	0.4
H15	−0.3450	0.7658	0.0034	0.072*	0.4
C11	−0.7216 (7)	0.7719 (3)	0.0321 (2)	0.0624 (10)	0.6
H16	−0.8340	0.7766	0.0728	0.075*	0.6
H17	−0.7987	0.7684	−0.0159	0.075*	0.6
C12	−0.5953 (5)	0.68650 (16)	0.05909 (12)	0.0624 (6)	
H18	−0.4697	0.6872	0.0223	0.075*	0.6
H19	−0.6855	0.6342	0.0546	0.075*	0.6
H20	−0.5686	0.6304	0.0375	0.075*	0.4
H21	−0.7554	0.6976	0.0628	0.075*	0.4
C13	−0.5062 (4)	0.66231 (13)	0.14222 (10)	0.0439 (5)	
C14	−0.3006 (4)	0.61736 (13)	0.15950 (11)	0.0465 (5)	
C15	−0.2239 (3)	0.60393 (13)	0.23604 (12)	0.0458 (5)	
H22	−0.0810	0.5762	0.2473	0.055*	
C16	−0.3447 (3)	0.62880 (13)	0.29652 (11)	0.0438 (5)	
C17	−0.5504 (3)	0.67436 (13)	0.27882 (11)	0.0410 (4)	
C18	−0.6270 (3)	0.68750 (13)	0.20271 (11)	0.0409 (4)	
H23	−0.7698	0.7152	0.1915	0.049*	
C19	−0.2635 (4)	0.6006 (2)	0.38046 (13)	0.0730 (8)	
H24	−0.1814	0.6546	0.3865	0.088*	0.5
H25	−0.3961	0.6001	0.4141	0.088*	0.5
H26	−0.3104	0.6488	0.4092	0.088*	0.5
H27	−0.1022	0.5927	0.3812	0.088*	0.5
C20	−0.3775 (7)	0.4969 (3)	0.4213 (2)	0.0438 (9)	0.5
H28	−0.4192	0.4961	0.4765	0.053*	0.5
H29	−0.5135	0.4949	0.3929	0.053*	0.5
C21	−0.1416 (7)	0.5230 (3)	0.4162 (2)	0.0446 (9)	0.5
H30	−0.1111	0.5271	0.4704	0.054*	0.5
H31	0.0001	0.5235	0.3876	0.054*	0.5
C22	−0.2538 (4)	0.4179 (2)	0.42155 (14)	0.0656 (7)	
H32	−0.2123	0.3718	0.4713	0.088 (18)*	0.5
H33	−0.4149	0.4262	0.4200	0.058 (13)*	0.5
H34	−0.3369	0.3655	0.4537	0.060 (13)*	0.5
H35	−0.1237	0.4216	0.4526	0.078 (15)*	0.5
C23	−0.1580 (5)	0.57678 (16)	0.10252 (13)	0.0699 (8)	
H36	−0.0159	0.5545	0.1277	0.084*	
H37	−0.1295	0.6282	0.0553	0.084*	
C24	−0.2510 (5)	0.49556 (16)	0.07572 (12)	0.0804 (9)	
H38	−0.1332	0.4667	0.0483	0.097*	
H39	−0.3658	0.5222	0.0367	0.097*	
C25	−0.3485 (5)	0.41715 (15)	0.14020 (12)	0.0644 (7)	
H40	−0.4924	0.4407	0.1565	0.077*	
H41	−0.3737	0.3632	0.1175	0.077*	
C26	−0.2098 (3)	0.38177 (13)	0.21302 (10)	0.0435 (5)	
C27	−0.0039 (3)	0.33628 (13)	0.21292 (11)	0.0448 (5)	
C28	0.1172 (3)	0.31807 (13)	0.28151 (12)	0.0443 (5)	
H42	0.2606	0.2902	0.2807	0.053*	
C29	0.0400 (3)	0.33839 (12)	0.35121 (10)	0.0392 (4)	
C30	−0.1672 (3)	0.38350 (12)	0.35142 (10)	0.0377 (4)	
C31	−0.2876 (3)	0.40213 (12)	0.28279 (11)	0.0396 (4)	
H43	−0.4307	0.4303	0.2835	0.048*	
C32	0.1769 (4)	0.30603 (15)	0.42464 (13)	0.0553 (6)	
H44	0.1901	0.3602	0.4484	0.066*	
H45	0.3255	0.2868	0.4090	0.066*	
C33	0.0864 (4)	0.22411 (16)	0.48715 (13)	0.0651 (7)	
H46	0.1605	0.2208	0.5373	0.078*	
H47	−0.0703	0.2395	0.4953	0.078*	
C34	0.1071 (4)	0.12618 (16)	0.47135 (15)	0.0628 (6)	
H48	0.2628	0.1108	0.4614	0.075*	
H49	0.0597	0.0795	0.5193	0.075*	
C35	−0.0238 (3)	0.11599 (13)	0.40259 (15)	0.0532 (6)	
C36	0.0739 (3)	0.11169 (14)	0.33139 (15)	0.0553 (6)	
H50	0.2286	0.1072	0.3271	0.066*	
C37	−0.0508 (4)	0.11380 (15)	0.26629 (16)	0.0595 (6)	
C38	−0.2743 (4)	0.11317 (17)	0.27859 (18)	0.0680 (7)	
H51	−0.3623	0.1133	0.2348	0.082*	
C39	−0.2509 (3)	0.11535 (15)	0.40745 (16)	0.0597 (6)	
H52	−0.3199	0.1171	0.4563	0.072*	
C40	0.0508 (5)	0.12235 (19)	0.18544 (18)	0.0774 (8)	
H53	−0.0669	0.1207	0.1484	0.093*	0.6
H54	0.1502	0.0661	0.1876	0.093*	0.6
H55	0.2112	0.1122	0.1902	0.093*	0.4
H56	−0.0033	0.0728	0.1624	0.093*	0.4
C41	0.1840 (8)	0.2138 (3)	0.1500 (2)	0.0668 (11)	0.6
H57	0.3133	0.2097	0.1834	0.080*	0.6
H58	0.2389	0.2097	0.0972	0.080*	0.6
C42	0.0035 (11)	0.2064 (4)	0.1380 (4)	0.0637 (16)	0.4
H59	−0.1577	0.2140	0.1391	0.076*	0.4
H60	0.0441	0.1999	0.0844	0.076*	0.4
C43	0.0848 (5)	0.30389 (18)	0.14163 (14)	0.0708 (7)	
H61	−0.0378	0.3097	0.1049	0.085*	0.6
H62	0.1923	0.3497	0.1150	0.085*	0.6
H63	0.2463	0.2999	0.1430	0.085*	0.4
H64	0.0403	0.3516	0.0932	0.085*	0.4

### Atomic displacement parameters (Å^2^)

**Table:**

	*U*^11^	*U*^22^	*U*^33^	*U*^12^	*U*^13^	*U*^23^
N1	0.0479 (11)	0.0539 (12)	0.1144 (18)	−0.0067 (9)	0.0227 (11)	−0.0285 (12)
N2	0.0409 (10)	0.0638 (12)	0.1137 (18)	−0.0058 (9)	−0.0037 (11)	−0.0448 (12)
C1	0.0666 (14)	0.0559 (13)	0.0480 (11)	−0.0095 (11)	0.0150 (10)	−0.0248 (10)
C2	0.0778 (15)	0.0628 (14)	0.0530 (12)	−0.0038 (12)	0.0063 (11)	−0.0346 (11)
C3	0.0511 (12)	0.0500 (12)	0.0799 (15)	−0.0006 (9)	0.0029 (11)	−0.0369 (12)
C4	0.0444 (10)	0.0324 (10)	0.0750 (14)	−0.0021 (8)	0.0053 (10)	−0.0234 (10)
C5	0.0450 (11)	0.0454 (12)	0.0942 (17)	−0.0045 (9)	−0.0016 (11)	−0.0292 (12)
C6	0.0770 (17)	0.0441 (13)	0.0855 (18)	−0.0014 (11)	0.0254 (14)	−0.0165 (12)
C7	0.0746 (15)	0.0308 (10)	0.0663 (14)	0.0007 (10)	0.0092 (12)	−0.0069 (9)
C8	0.0471 (11)	0.0335 (10)	0.0720 (14)	−0.0005 (8)	−0.0029 (10)	−0.0155 (10)
C9	0.137 (3)	0.0452 (13)	0.0563 (14)	0.0068 (15)	−0.0120 (15)	−0.0034 (11)
C10	0.091 (4)	0.048 (3)	0.037 (3)	−0.008 (3)	−0.003 (3)	−0.002 (2)
C11	0.093 (3)	0.051 (2)	0.0446 (19)	0.011 (2)	−0.0237 (19)	−0.0144 (16)
C12	0.1004 (19)	0.0522 (13)	0.0364 (11)	−0.0181 (12)	−0.0042 (11)	−0.0109 (9)
C13	0.0676 (13)	0.0327 (9)	0.0324 (9)	−0.0112 (9)	0.0030 (8)	−0.0089 (7)
C14	0.0672 (13)	0.0309 (9)	0.0396 (10)	−0.0050 (9)	0.0170 (9)	−0.0068 (8)
C15	0.0492 (11)	0.0328 (10)	0.0515 (11)	−0.0034 (8)	0.0065 (9)	−0.0036 (8)
C16	0.0559 (11)	0.0379 (10)	0.0379 (10)	−0.0118 (9)	−0.0004 (8)	−0.0080 (8)
C17	0.0536 (11)	0.0360 (9)	0.0370 (9)	−0.0121 (8)	0.0084 (8)	−0.0147 (8)
C18	0.0478 (10)	0.0337 (9)	0.0434 (10)	−0.0084 (8)	0.0026 (8)	−0.0123 (8)
C19	0.0617 (14)	0.105 (2)	0.0419 (12)	−0.0207 (14)	−0.0051 (10)	0.0048 (12)
C20	0.065 (2)	0.041 (2)	0.0260 (16)	0.0018 (18)	0.0066 (16)	−0.0124 (15)
C21	0.062 (2)	0.043 (2)	0.0361 (19)	−0.0004 (18)	−0.0067 (17)	−0.0238 (16)
C22	0.0569 (13)	0.101 (2)	0.0585 (14)	−0.0181 (13)	0.0111 (11)	−0.0554 (15)
C23	0.105 (2)	0.0451 (12)	0.0507 (13)	0.0090 (12)	0.0363 (13)	−0.0029 (10)
C24	0.159 (3)	0.0534 (13)	0.0282 (10)	0.0263 (16)	0.0051 (13)	−0.0161 (10)
C25	0.110 (2)	0.0449 (12)	0.0435 (12)	0.0036 (12)	−0.0247 (12)	−0.0202 (10)
C26	0.0670 (13)	0.0339 (9)	0.0330 (9)	−0.0082 (9)	−0.0032 (8)	−0.0133 (7)
C27	0.0641 (12)	0.0366 (10)	0.0388 (10)	−0.0132 (9)	0.0129 (9)	−0.0181 (8)
C28	0.0466 (10)	0.0359 (10)	0.0554 (12)	−0.0076 (8)	0.0077 (9)	−0.0202 (9)
C29	0.0490 (10)	0.0314 (9)	0.0393 (10)	−0.0099 (8)	−0.0017 (8)	−0.0108 (8)
C30	0.0502 (10)	0.0349 (9)	0.0322 (9)	−0.0092 (8)	0.0044 (7)	−0.0151 (7)
C31	0.0488 (10)	0.0334 (9)	0.0401 (10)	−0.0028 (8)	−0.0016 (8)	−0.0153 (8)
C32	0.0626 (13)	0.0455 (11)	0.0574 (13)	−0.0127 (10)	−0.0183 (10)	−0.0080 (10)
C33	0.0859 (17)	0.0526 (13)	0.0535 (13)	−0.0114 (12)	−0.0261 (12)	−0.0020 (10)
C34	0.0543 (13)	0.0446 (12)	0.0803 (16)	−0.0028 (10)	−0.0135 (11)	0.0036 (11)
C35	0.0433 (11)	0.0291 (9)	0.0860 (16)	−0.0004 (8)	−0.0084 (10)	−0.0114 (10)
C36	0.0391 (10)	0.0331 (10)	0.0970 (17)	0.0025 (8)	0.0045 (11)	−0.0240 (11)
C37	0.0520 (12)	0.0411 (11)	0.0972 (18)	−0.0018 (9)	−0.0017 (12)	−0.0399 (12)
C38	0.0522 (13)	0.0585 (14)	0.108 (2)	−0.0025 (11)	−0.0110 (13)	−0.0475 (14)
C39	0.0447 (11)	0.0446 (12)	0.0938 (17)	−0.0024 (9)	0.0033 (11)	−0.0253 (12)
C40	0.0780 (17)	0.0647 (17)	0.110 (2)	−0.0023 (13)	0.0105 (15)	−0.0612 (17)
C41	0.084 (3)	0.069 (3)	0.050 (2)	0.018 (2)	0.014 (2)	−0.0262 (19)
C42	0.071 (4)	0.064 (4)	0.075 (4)	−0.011 (3)	0.022 (3)	−0.053 (3)
C43	0.0954 (18)	0.0707 (16)	0.0608 (14)	−0.0262 (14)	0.0334 (13)	−0.0428 (13)

### Geometric parameters (Å, º)

**Table:**

N1—C5	1.327 (3)	C21—H30	0.9900
N1—C6	1.342 (3)	C21—H31	0.9900
N2—C39	1.332 (3)	C22—C30	1.512 (2)
N2—C38	1.341 (3)	C22—H32	0.9900
C1—C17	1.518 (2)	C22—H33	0.9900
C1—C2	1.539 (3)	C22—H34	0.9900
C1—H1	0.9900	C22—H35	0.9900
C1—H2	0.9900	C23—C24	1.527 (4)
C2—C3	1.524 (3)	C23—H36	0.9900
C2—H3	0.9900	C23—H37	0.9900
C2—H4	0.9900	C24—C25	1.532 (3)
C3—C4	1.507 (3)	C24—H38	0.9900
C3—H5	0.9900	C24—H39	0.9900
C3—H6	0.9900	C25—C26	1.518 (3)
C4—C8	1.378 (3)	C25—H40	0.9900
C4—C5	1.399 (3)	C25—H41	0.9900
C5—H7	0.9500	C26—C31	1.394 (2)
C6—C7	1.383 (4)	C26—C27	1.395 (3)
C6—H8	0.9500	C27—C28	1.392 (3)
C7—C8	1.381 (3)	C27—C43	1.519 (2)
C7—C9	1.507 (4)	C28—C29	1.391 (3)
C8—H9	0.9500	C28—H42	0.9500
C9—C10	1.544 (6)	C29—C30	1.399 (3)
C9—C11	1.621 (5)	C29—C32	1.516 (3)
C9—H10	0.9900	C30—C31	1.390 (2)
C9—H11	0.9900	C31—H43	0.9500
C9—H12	0.9900	C32—C33	1.528 (3)
C9—H13	0.9900	C32—H44	0.9900
C10—C12	1.512 (6)	C32—H45	0.9900
C10—H14	0.9900	C33—C34	1.524 (3)
C10—H15	0.9900	C33—H46	0.9900
C11—C12	1.416 (4)	C33—H47	0.9900
C11—H16	0.9900	C34—C35	1.509 (3)
C11—H17	0.9900	C34—H48	0.9900
C12—C13	1.521 (3)	C34—H49	0.9900
C12—H18	0.9900	C35—C36	1.383 (3)
C12—H19	0.9900	C35—C39	1.395 (3)
C12—H20	0.9900	C36—C37	1.385 (3)
C12—H21	0.9900	C36—H50	0.9500
C13—C18	1.388 (3)	C37—C38	1.383 (3)
C13—C14	1.395 (3)	C37—C40	1.510 (4)
C14—C15	1.397 (3)	C38—H51	0.9500
C14—C23	1.517 (3)	C39—H52	0.9500
C15—C16	1.386 (3)	C40—C42	1.325 (7)
C15—H22	0.9500	C40—C41	1.591 (5)
C16—C17	1.399 (3)	C40—H53	0.9900
C16—C19	1.516 (3)	C40—H54	0.9900
C17—C18	1.391 (3)	C40—H55	0.9900
C18—H23	0.9500	C40—H56	0.9900
C19—C21	1.350 (5)	C41—C43	1.396 (5)
C19—C20	1.691 (5)	C41—H57	0.9900
C19—H24	0.9900	C41—H58	0.9900
C19—H25	0.9900	C42—C43	1.555 (6)
C19—H26	0.9900	C42—H59	0.9900
C19—H27	0.9900	C42—H60	0.9900
C20—C22	1.347 (4)	C43—H61	0.9900
C20—H28	0.9900	C43—H62	0.9900
C20—H29	0.9900	C43—H63	0.9900
C21—C22	1.701 (5)	C43—H64	0.9900
			
C5—N1—C6	116.8 (2)	C30—C22—H32	110.3
C39—N2—C38	116.6 (2)	C21—C22—H32	110.3
C17—C1—C2	114.26 (18)	C30—C22—H33	110.3
C17—C1—H1	108.7	C21—C22—H33	110.3
C2—C1—H1	108.7	H32—C22—H33	108.6
C17—C1—H2	108.7	C20—C22—H34	105.3
C2—C1—H2	108.7	C30—C22—H34	105.3
H1—C1—H2	107.6	C20—C22—H35	105.3
C3—C2—C1	117.46 (19)	C30—C22—H35	105.3
C3—C2—H3	107.9	H34—C22—H35	106.0
C1—C2—H3	107.9	C14—C23—C24	115.7 (2)
C3—C2—H4	107.9	C14—C23—H36	108.4
C1—C2—H4	107.9	C24—C23—H36	108.4
H3—C2—H4	107.2	C14—C23—H37	108.4
C4—C3—C2	114.39 (17)	C24—C23—H37	108.4
C4—C3—H5	108.7	H36—C23—H37	107.4
C2—C3—H5	108.7	C23—C24—C25	116.52 (17)
C4—C3—H6	108.7	C23—C24—H38	108.2
C2—C3—H6	108.7	C25—C24—H38	108.2
H5—C3—H6	107.6	C23—C24—H39	108.2
C8—C4—C5	116.5 (2)	C25—C24—H39	108.2
C8—C4—C3	122.13 (19)	H38—C24—H39	107.3
C5—C4—C3	121.2 (2)	C26—C25—C24	115.1 (2)
N1—C5—C4	124.2 (2)	C26—C25—H40	108.5
N1—C5—H7	117.9	C24—C25—H40	108.5
C4—C5—H7	117.9	C26—C25—H41	108.5
N1—C6—C7	124.4 (2)	C24—C25—H41	108.5
N1—C6—H8	117.8	H40—C25—H41	107.5
C7—C6—H8	117.8	C31—C26—C27	118.34 (17)
C8—C7—C6	116.8 (2)	C31—C26—C25	117.50 (19)
C8—C7—C9	121.8 (2)	C27—C26—C25	124.01 (18)
C6—C7—C9	121.3 (2)	C28—C27—C26	118.03 (16)
C4—C8—C7	121.1 (2)	C28—C27—C43	120.2 (2)
C4—C8—H9	119.4	C26—C27—C43	121.71 (19)
C7—C8—H9	119.4	C29—C28—C27	123.65 (18)
C7—C9—C10	109.2 (3)	C29—C28—H42	118.2
C7—C9—C11	113.0 (2)	C27—C28—H42	118.2
C7—C9—H10	109.0	C28—C29—C30	118.22 (17)
C11—C9—H10	109.0	C28—C29—C32	118.96 (18)
C7—C9—H11	109.0	C30—C29—C32	122.72 (17)
C11—C9—H11	109.0	C31—C30—C29	118.05 (16)
H10—C9—H11	107.8	C31—C30—C22	120.01 (18)
C7—C9—H12	109.8	C29—C30—C22	121.79 (17)
C10—C9—H12	109.8	C30—C31—C26	123.58 (18)
C7—C9—H13	109.8	C30—C31—H43	118.2
C10—C9—H13	109.8	C26—C31—H43	118.2
H12—C9—H13	108.3	C29—C32—C33	114.53 (17)
C12—C10—C9	115.1 (4)	C29—C32—H44	108.6
C12—C10—H14	108.5	C33—C32—H44	108.6
C9—C10—H14	108.5	C29—C32—H45	108.6
C12—C10—H15	108.5	C33—C32—H45	108.6
C9—C10—H15	108.5	H44—C32—H45	107.6
H14—C10—H15	107.5	C34—C33—C32	117.7 (2)
C12—C11—C9	116.1 (3)	C34—C33—H46	107.9
C12—C11—H16	108.3	C32—C33—H46	107.9
C9—C11—H16	108.3	C34—C33—H47	107.9
C12—C11—H17	108.3	C32—C33—H47	107.9
C9—C11—H17	108.3	H46—C33—H47	107.2
H16—C11—H17	107.4	C35—C34—C33	114.42 (17)
C11—C12—C13	119.1 (2)	C35—C34—H48	108.7
C10—C12—C13	117.6 (3)	C33—C34—H48	108.7
C11—C12—H18	107.5	C35—C34—H49	108.7
C13—C12—H18	107.5	C33—C34—H49	108.7
C11—C12—H19	107.5	H48—C34—H49	107.6
C13—C12—H19	107.5	C36—C35—C39	117.3 (2)
H18—C12—H19	107.0	C36—C35—C34	121.9 (2)
C10—C12—H20	107.9	C39—C35—C34	120.8 (2)
C13—C12—H20	107.9	C35—C36—C37	120.85 (19)
C10—C12—H21	107.9	C35—C36—H50	119.6
C13—C12—H21	107.9	C37—C36—H50	119.6
H20—C12—H21	107.2	C38—C37—C36	116.2 (2)
C18—C13—C14	118.27 (17)	C38—C37—C40	121.9 (2)
C18—C13—C12	120.13 (19)	C36—C37—C40	121.8 (2)
C14—C13—C12	121.57 (18)	N2—C38—C37	125.2 (2)
C13—C14—C15	118.04 (17)	N2—C38—H51	117.4
C13—C14—C23	124.8 (2)	C37—C38—H51	117.4
C15—C14—C23	117.1 (2)	N2—C39—C35	123.7 (2)
C16—C15—C14	123.64 (19)	N2—C39—H52	118.1
C16—C15—H22	118.2	C35—C39—H52	118.1
C14—C15—H22	118.2	C42—C40—C37	111.4 (3)
C15—C16—C17	118.06 (17)	C37—C40—C41	116.6 (2)
C15—C16—C19	120.7 (2)	C37—C40—H53	108.1
C17—C16—C19	121.05 (19)	C41—C40—H53	108.1
C18—C17—C16	118.22 (17)	C37—C40—H54	108.1
C18—C17—C1	118.57 (18)	C41—C40—H54	108.1
C16—C17—C1	123.10 (17)	H53—C40—H54	107.3
C13—C18—C17	123.63 (18)	C42—C40—H55	109.3
C13—C18—H23	118.2	C37—C40—H55	109.3
C17—C18—H23	118.2	C42—C40—H56	109.3
C21—C19—C16	128.5 (3)	C37—C40—H56	109.3
C16—C19—C20	104.8 (2)	H55—C40—H56	108.0
C21—C19—H24	105.2	C43—C41—C40	120.6 (3)
C16—C19—H24	105.2	C43—C41—H57	107.2
C21—C19—H25	105.2	C40—C41—H57	107.2
C16—C19—H25	105.2	C43—C41—H58	107.2
H24—C19—H25	105.9	C40—C41—H58	107.2
C16—C19—H26	110.8	H57—C41—H58	106.8
C20—C19—H26	110.8	C40—C42—C43	128.5 (5)
C16—C19—H27	110.8	C40—C42—H59	105.2
C20—C19—H27	110.8	C43—C42—H59	105.2
H26—C19—H27	108.9	C40—C42—H60	105.2
C22—C20—C19	116.5 (3)	C43—C42—H60	105.2
C22—C20—H28	108.2	H59—C42—H60	105.9
C19—C20—H28	108.2	C41—C43—C27	120.9 (3)
C22—C20—H29	108.2	C27—C43—C42	113.0 (3)
C19—C20—H29	108.2	C41—C43—H61	107.1
H28—C20—H29	107.3	C27—C43—H61	107.1
C19—C21—C22	115.7 (3)	C41—C43—H62	107.1
C19—C21—H30	108.4	C27—C43—H62	107.1
C22—C21—H30	108.4	H61—C43—H62	106.8
C19—C21—H31	108.4	C27—C43—H63	109.0
C22—C21—H31	108.4	C42—C43—H63	109.0
H30—C21—H31	107.4	C27—C43—H64	109.0
C20—C22—C30	127.9 (3)	C42—C43—H64	109.0
C30—C22—C21	106.9 (2)	H63—C43—H64	107.8
			
C17—C1—C2—C3	75.5 (3)	C19—C21—C22—C30	94.6 (3)
C1—C2—C3—C4	−66.0 (3)	C13—C14—C23—C24	−63.4 (3)
C2—C3—C4—C8	107.4 (2)	C15—C14—C23—C24	113.0 (2)
C2—C3—C4—C5	−69.0 (3)	C14—C23—C24—C25	−46.4 (3)
C6—N1—C5—C4	−1.6 (3)	C23—C24—C25—C26	−46.4 (3)
C8—C4—C5—N1	−1.8 (3)	C24—C25—C26—C31	111.4 (2)
C3—C4—C5—N1	174.8 (2)	C24—C25—C26—C27	−63.9 (3)
C5—N1—C6—C7	1.9 (4)	C31—C26—C27—C28	−3.2 (3)
N1—C6—C7—C8	1.2 (3)	C25—C26—C27—C28	172.08 (18)
N1—C6—C7—C9	−174.4 (2)	C31—C26—C27—C43	174.58 (18)
C5—C4—C8—C7	5.0 (3)	C25—C26—C27—C43	−10.1 (3)
C3—C4—C8—C7	−171.58 (17)	C26—C27—C28—C29	3.4 (3)
C6—C7—C8—C4	−4.8 (3)	C43—C27—C28—C29	−174.48 (18)
C9—C7—C8—C4	170.89 (19)	C27—C28—C29—C30	−3.0 (3)
C8—C7—C9—C10	−117.0 (3)	C27—C28—C29—C32	173.57 (17)
C6—C7—C9—C10	58.5 (4)	C28—C29—C30—C31	2.5 (3)
C8—C7—C9—C11	−59.7 (3)	C32—C29—C30—C31	−173.94 (16)
C6—C7—C9—C11	115.8 (3)	C28—C29—C30—C22	−173.11 (19)
C7—C9—C10—C12	73.5 (5)	C32—C29—C30—C22	10.5 (3)
C11—C9—C10—C12	−31.4 (3)	C20—C22—C30—C31	−32.6 (4)
C7—C9—C11—C12	−63.3 (4)	C21—C22—C30—C31	−95.1 (2)
C10—C9—C11—C12	34.1 (3)	C20—C22—C30—C29	142.9 (3)
C9—C11—C12—C10	−32.8 (3)	C21—C22—C30—C29	80.4 (3)
C9—C11—C12—C13	73.3 (4)	C29—C30—C31—C26	−2.7 (3)
C9—C10—C12—C11	34.3 (4)	C22—C30—C31—C26	173.01 (19)
C9—C10—C12—C13	−74.3 (5)	C27—C26—C31—C30	3.1 (3)
C11—C12—C13—C18	35.0 (4)	C25—C26—C31—C30	−172.56 (18)
C10—C12—C13—C18	101.7 (4)	C28—C29—C32—C33	−108.0 (2)
C11—C12—C13—C14	−143.1 (3)	C30—C29—C32—C33	68.4 (3)
C10—C12—C13—C14	−76.3 (4)	C29—C32—C33—C34	73.9 (3)
C18—C13—C14—C15	−2.7 (3)	C32—C33—C34—C35	−65.6 (3)
C12—C13—C14—C15	175.31 (18)	C33—C34—C35—C36	105.7 (2)
C18—C13—C14—C23	173.67 (19)	C33—C34—C35—C39	−71.0 (3)
C12—C13—C14—C23	−8.3 (3)	C39—C35—C36—C37	4.7 (3)
C13—C14—C15—C16	3.1 (3)	C34—C35—C36—C37	−172.15 (18)
C23—C14—C15—C16	−173.58 (18)	C35—C36—C37—C38	−4.7 (3)
C14—C15—C16—C17	−3.5 (3)	C35—C36—C37—C40	171.67 (19)
C14—C15—C16—C19	171.89 (19)	C39—N2—C38—C37	2.1 (4)
C15—C16—C17—C18	3.5 (3)	C36—C37—C38—N2	1.3 (3)
C19—C16—C17—C18	−171.89 (19)	C40—C37—C38—N2	−175.1 (2)
C15—C16—C17—C1	−172.74 (18)	C38—N2—C39—C35	−2.2 (3)
C19—C16—C17—C1	11.9 (3)	C36—C35—C39—N2	−1.1 (3)
C2—C1—C17—C18	−106.2 (2)	C34—C35—C39—N2	175.7 (2)
C2—C1—C17—C16	69.9 (3)	C38—C37—C40—C42	67.1 (4)
C14—C13—C18—C17	3.1 (3)	C36—C37—C40—C42	−109.1 (4)
C12—C13—C18—C17	−174.99 (17)	C38—C37—C40—C41	116.7 (3)
C16—C17—C18—C13	−3.4 (3)	C36—C37—C40—C41	−59.5 (4)
C1—C17—C18—C13	172.93 (17)	C42—C40—C41—C43	37.8 (4)
C15—C16—C19—C21	−31.6 (4)	C37—C40—C41—C43	−56.7 (5)
C17—C16—C19—C21	143.6 (3)	C37—C40—C42—C43	69.5 (6)
C15—C16—C19—C20	−92.8 (2)	C41—C40—C42—C43	−37.3 (4)
C17—C16—C19—C20	82.4 (3)	C40—C41—C43—C27	61.4 (5)
C21—C19—C20—C22	−30.3 (3)	C40—C41—C43—C42	−31.4 (4)
C16—C19—C20—C22	96.1 (3)	C28—C27—C43—C41	43.8 (4)
C16—C19—C21—C22	−60.6 (4)	C26—C27—C43—C41	−133.9 (3)
C20—C19—C21—C22	23.4 (2)	C28—C27—C43—C42	94.5 (4)
C19—C20—C22—C30	−63.5 (4)	C26—C27—C43—C42	−83.3 (4)
C19—C20—C22—C21	23.7 (2)	C40—C42—C43—C41	43.5 (5)
C19—C21—C22—C20	−29.9 (3)	C40—C42—C43—C27	−67.9 (6)

### Hydrogen-bond geometry (Å, º)

**Table:**

*D*—H···*A*	*D*—H	H···*A*	*D*···*A*	*D*—H···*A*
C8—H9···N1^i^	0.95	2.43	3.373 (3)	173
C36—H50···N2^ii^	0.95	2.47	3.394 (3)	165

Symmetry codes: (i) *x*−1, *y*, *z*; (ii) *x*+1, *y*, *z*.
